# Is in vitro cytokine release a suitable marker to improve the diagnosis of suspected mold-related respiratory symptoms? A proof-of-concept study 

**DOI:** 10.5414/ALX02299E

**Published:** 2022-03-29

**Authors:** Verena Liebers, Sabine Kespohl, Gerda Borowitzki, Heike Stubel, Monika Raulf

**Affiliations:** Institute for Prevention and Occupational Medicine of the German Social Accident Insurance, Institute of the Ruhr University Bochum (IPA), Germany

**Keywords:** cytokine release, endotoxin, mold, mold exposure, respiratory symptoms, whole blood assay

## Abstract

Indoor mold infestation can lead to a variety of adverse health effects, including allergic and non-allergic respiratory complaints. Especially if no evidence of an allergic reaction can be found for the complaints, diagnostic tools that might explain mold-associated health problems are missing. As a proof-of-concept, in the present study whole blood assay (WBA) was used to determine cellular response by measuring cytokine release (IL-1β and IL-8) after in vitro stimulation. Blood was available from a total of 48 subjects. By questionnaire, complaints and possible mold exposure were documented. Specific in vitro blood stimulation was tested with *Escherichia coli* endotoxin and extracts of different molds (*Aspergillus fumigatus*, *Penicillium chrysogenum*, *Aspergillus versicolor*, and *Cladosporium herbarum*). To characterize the relevance of WBA in describing the mold-induced immune response, we compared the following groups: asthmatics vs. non-asthmatics, mx1-sensitized vs. non-mx1-sensitized, mold-exposed vs. non-mold-exposed. In response to endotoxin stimulation, a significantly higher IL-1β release was found in mx1-sensitized than in non-mx1-sensitized subjects. Furthermore, the blood of asthmatics showed significantly higher IL-8 and IL-1β release after stimulation with *Penicillium chrysogenum* and endotoxin, respectively, compared to non-asthmatics. However, no significant difference in the level of cytokine release was observed between the mold-exposed and non-exposed group, neither after endotoxin nor mold stimulation. In conclusion, the WBA used in this study is not a suitable tool for clinical routine diagnostic workup. Our data suggests that WBA reflects cellular differences that are disease-related but not directly attributable to mold exposure. However, in combination with further data, WBA will be a helpful und interesting tool in research, e.g., in description of the complex immune response to molds.

## Introduction 

Health effects due to mold exposure are widely discussed, especially in the field of preventive health care [1]. Fungal exposure and/or sensitization to molds has been shown to be strongly associated with the development of asthma and allergic airway diseases [2, 3]. However, such associations are not as prevalent as previously suggested [4]. In general, inhalation of airborne fungal particles can cause a wide variety of symptoms, and the World Health Organization guideline [5] documented that occupants of damp indoor environments are at risk for upper and lower respiratory symptoms [6]. However, studies showing clear dose-response relationships regarding mold exposure and health effects are scarce and difficult to evaluate. Firstly, molds are only one part of the complex bioaerosol burden, and secondly, a serological determination of mold-specific IgE, for example, does not clarify all mold-associated complaints. Consequently, there is a need for further reliable and reproducible methods for the determination of immunoreactivity due to mold exposure, taking individual heterogeneity into account. 

Cellular analysis has recently gained attention as a possible additional tool to diagnose immunoreactivity due to mold exposure [2, 7]. In particular, direct determination through the use of whole blood represents one approach to gain insight into the immune response, combined with the advantage of relatively simple sample handling [7]. In general, the whole blood assay (WBA) may be used in two different ways: with a cryopreserved pooled blood sample that is composed of blood from several donors as a tool to describe pyrogenic and proinflammatory characteristics of bioaerosol/dust samples (stress measurement), or with fresh blood to analyze changes in the reactivity of an individual (strain measurement). 

In one of our pilot studies to characterize individual strain measurement using the WBA, we identified an enhanced number of blood leucocytes as well as increased expression of Toll-like receptor (TLR)-2, TLR-4, and dectin-1 on the cell surface of blood monocytes from subjects exposed to moisture damage compared to respective controls who lived in houses without visible moisture damage [8]. The release of IL-8 and IL-1β from whole blood was increased after in vitro stimulation with extract from *Aspergillus versicolor* in subjects exposed to moisture damage. For these moisture-exposed subjects, IL-1β release was also significantly enhanced after in vitro stimulation with *Escherichia coli* endotoxin [9]. These results indicated that mold-induced immune reactions can be detected by WBA, but need further validation. 

In the case of an immune response to molds, it must be considered that such responses can be induced by various fungal components, such as β-glucans, chitins, and proteases, which directly activate the innate immune system involving pathogen-associated molecular patterns (PAMPs) and their counterparts, the pattern recognition receptors (PRRs) [2, 10, 11]. 

The aim of our study was to describe mold-exposed and non-exposed subjects in terms of humoral acquired [12] and innate immunity. The WBA was used to measure cytokine release after in vitro stimulation with endotoxin or mold extracts, and to investigate the association with exposure and questionnaire-recorded symptoms. This proof-of-concept study is intended to illuminate aspects of the complex interaction between mold exposure and the innate immune system, as well as the humoral immune response. Furthermore, this study investigates the utility of the WBA in the context of recording mold exposure. 

## Materials and methods 

### Study group and study design 

The study was approved by the Ethics committee of the Ruhr-University Bochum, Germany, (registration number: 4231-12) and performed in accordance with the Declaration of Helsinki. All participants gave their written approval for participation. In total, 69 subjects were enrolled in the current study (characterization in [12]), and the WBA was performed in 48 subjects for the proof-of-concept study. 

A detailed questionnaire was used to collect data from the study participants on their indoor mold exposure and dampness at their homes and workplaces, including questions about extent and duration of mold and dampness exposure. Based on the information obtained from the questionnaire, the study group was divided into mold-exposed and non-exposed individuals. 

### Whole blood assay 

The WBA is a two-step assay. In step 1, blood is incubated with the relevant stimuli, and in step 2, cytokine release is quantified in the cell-free supernatant. Whole blood was available from 48 study participants. Heparinized venous blood was collected and processed promptly for the WBA, which was performed according to standardized protocols with different endotoxin concentrations and mold extracts, followed by quantification of the release of IL-1β and IL-8 into the cell-free supernatant [described in detail in [8, 13, 14]. 

Briefly, blood was incubated with endotoxin (or with RPMI 1640 medium as control) or with extracts from different molds (mold extract was produced as 30 mg lyophilized mold material/mL distilled water and used at a final concentration of 1 mg/mL in WBA). The incubation was performed in a total volume of 1,000 µL (100 µL blood + 100 µL stimulus + 800 µL RPMI) for 22 hours at 37 °C and 5% CO_2_. After centrifugation (2 minutes, 10,000 × g), cell-free supernatants were aliquoted and frozen at –80 °C until analysis. 

The released cytokines IL-1β and IL-8 were measured in the cell-free supernatant using enzyme-linked-immunosorbent assay (ELISA) kits (IL-1β, DuoSetTM; R&D Systems, Wiesbaden, Germany; IL-8: Becton Dickinson, Heidelberg, Germany), according to the recommendations of the manufacturers with a sensitivity range of 3.9 – 250 pg/mL for IL-1β, and 3 – 200 pg/mL for IL-8. 

All samples were measured in 2 – 3 different dilutions, and the results were accepted if the coefficient of variation (CV) was below 25%, otherwise the measurements were repeated. The background of cytokine release (spontaneous release of non-stimulated blood) was defined as the mean value of the group in non-stimulated samples plus twice the standard deviation. Thus, the backgrounds for IL-1β and IL-8 were set at 13.1 pg/mL and 450 pg/mL, respectively. 

### Stimuli and reagents 

The *Escherichia coli* O113:H10 control standard endotoxin (Haemochrom Diagnostica, Essen, Germany) was used in five different final concentrations (1, 10, 40, 100, 1,000 pg/mL) in the WBA. Four different molds were purchased from Allergon (Ängelholm, Sweden): *Aspergillus fumigatus* (Lot. 100906205), *Aspergillus versicolor* (Lot. 101507011), *Cladosporium herbarum* (Lot. 103409011), *Penicillium chrysogenum* (Lot.109070021) as freeze-dried mold substance. They were all weighed (30 mg per 1 mL distilled water), vortexed for 2 minutes and shaken in a 50-mL tube for 1 hour on a horizontal shaker. The mold extract was then autoclaved at 120 °C for 20 minutes (VARIOKLAV 400 S, Thermo Fisher Scientific, Oberschleißheim, Germany), centrifuged (3,000 × g, 10 minutes), and aliquots of the supernatant (150 µL portions each) were frozen at –20 °C. RPMI 1640 supplemented with glutamine and HEPES (both from Gibco, Thermo Fisher Scientific, Darmstadt, Germany) were used in all the WBA. 

### Serological analysis 

Serum was collected for the determination of total IgE and specific IgE to mold allergen mix (mx1 including *Penicillium chrysogenum*, *Cladosporium herbarum*, *Aspergillus fumigatus*, *Alternaria alternata*) using ImmunoCAP 250 (Thermo Fisher Scientific, Uppsala, Sweden). Furthermore, specific IgE against a mixture of ubiquitous environmental allergens (sx1, including *Dermatophagoides pteronyssinus*, cat, dog, timothy grass, rye, *Cladosporium herbarum*, birch, mugwort) were measured. Specific IgE values ≥ 0.35 kU/L were considered positive [12]. 

### Statistics 

Data were analyzed and graphs were made with GraphPad Prism, version 9.0 (GraphPad Software Inc., San Diego, CA, USA). Values below the detection limit were assigned corresponding to 2/3 of the detection limit. For the descriptive analysis, median, further percentiles, mean, standard deviation, and coefficient of variation (CV) were calculated. Statistical analysis was calculated with Mann-Whitney test or Wilcoxon test and Spearman correlation. p-values < 0.05 were considered statistically significant. 

## Results 

### Characterization of the study group and WBA according to mold exposure 

Based on the data obtained from the questionnaires [12], the 48 subjects were divided into two groups: mold-exposed (n = 29) or non-exposed (n = 19) (Table 1). 

Both groups were similar regarding gender, age, and smoking habits, but there were more positive serum results (≥ 0.35 kU/L) against mx1 and sx1 in the mold-exposed compared to the non-exposed group (mx1: 34 vs. 11%; sx1: 62 vs. 42%). Respiratory symptoms, especially asthma also occurred more frequently in the mold-exposed group. 

In WBA, IL-1β and IL-8 release were measured as outcome. Correlation of these two cytokines regarding the whole study group (n = 48) was highly significant (p < 0.0001, Spearmans rank correlation) upon stimulation with *Penicillium chrysogenum* (r_s_ = 0.82) and *Aspergillus fumigatus* (r_s_ = 0.80) and after stimulation with 10 pg/mL endotoxin (r_s_ = 0.55). 

**Figure 1.**
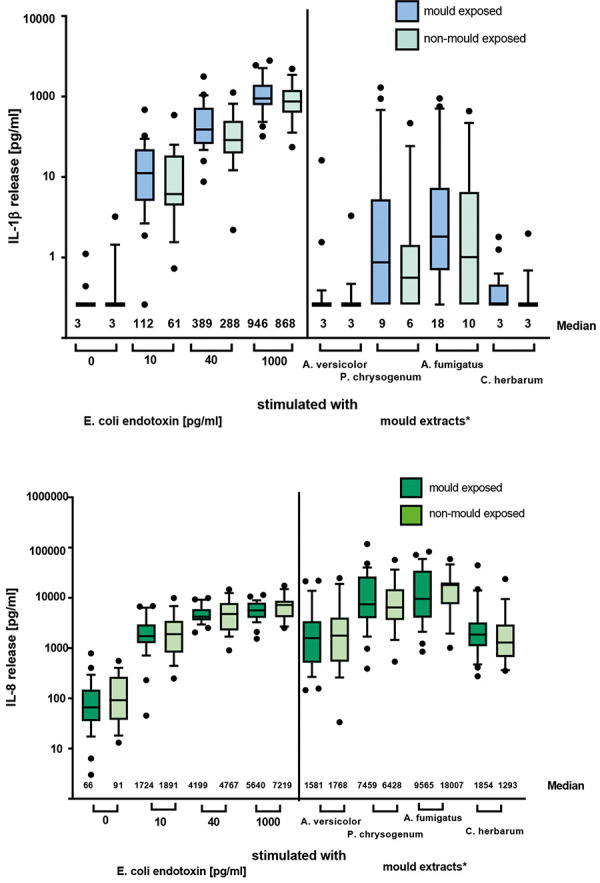
Comparison of cytokine release in whole blood assay from mold-exposed (n = 29) and non-exposed (n =19) subjects with respect to IL-1β and IL-8 release after stimulation with endotoxin or mold extracts. Median and 90% percentile are shown. *Molds were used in an estimated final concentration of 1 mg/mL based on calculation of the total extracted weight of mold (30 mg/mL).

Figure 1 shows the results of the IL-1β and IL-8 release in the mold-exposed and non-exposed groups after stimulation with endotoxin and mold extracts. A clear dose-dependent release of both cytokines was measured after stimulation with endotoxin in all subjects, independent of their mold exposure. The level of cytokine release varied greatly among subjects. Overall, the IL-1β release induced by endotoxin ranged from below the detection limit (2.6 pg/mL) to a maximum of 2,793 pg/mL. In unstimulated samples, spontaneous IL-1β release was between the detection limit and a maximum of 32.2 pg/mL. Compared to endotoxin, stimulation with mold extracts was less effective and reached a maximum IL-1β release of 1,293 pg/mL. Of the four mold extracts used, *Penicillium chrysogenum* induced the highest IL-1β release. There was no significant difference between mold-exposed and non-exposed group, independent of stimuli. However, 53% of the non-exposed subjects released IL-1β in response to any of the tested extracts, whereas 72% of the subjects in the mold-exposed group responded to the tested extracts. 

IL-8 release varied widely among the subjects, with spontaneous IL-8 release ranging from the detection limit (2 pg/mL) to 782 pg/mL. The maximum release reached values of up to 17,443 pg/mL after endotoxin stimulation and up to 117,742 pg/mL after mold stimulation. Furthermore, stimulation with *Aspergillus fumigatus* resulted in IL-8 release that was above the background levels in blood of all participants. 

No significant differences in IL-8 release was measured between the mold-exposed and non-exposed groups. However, despite no significant differences in both IL-8 and IL-1β release we detected a slightly lower cytokine release in the non-exposed compared to the mold-exposed subjects for both cytokines (Figure 1a, b). 

### Characterization of the study group and whole blood assay according to asthma symptoms 

According to the information provided in the questionnaire, we classified the study group into subjects with (n = 35) and without (n = 13) respiratory symptoms. 24 of the 35 subjects with respiratory symptoms had an asthma diagnosis. Thus, the WBA results from 24 asthmatic subjects were compared to those from 24 non-asthmatics (11 of whom reported respiratory symptoms). Characteristics of the asthmatic and non-asthmatic groups are presented in Table 2. 

**Figure 2.**
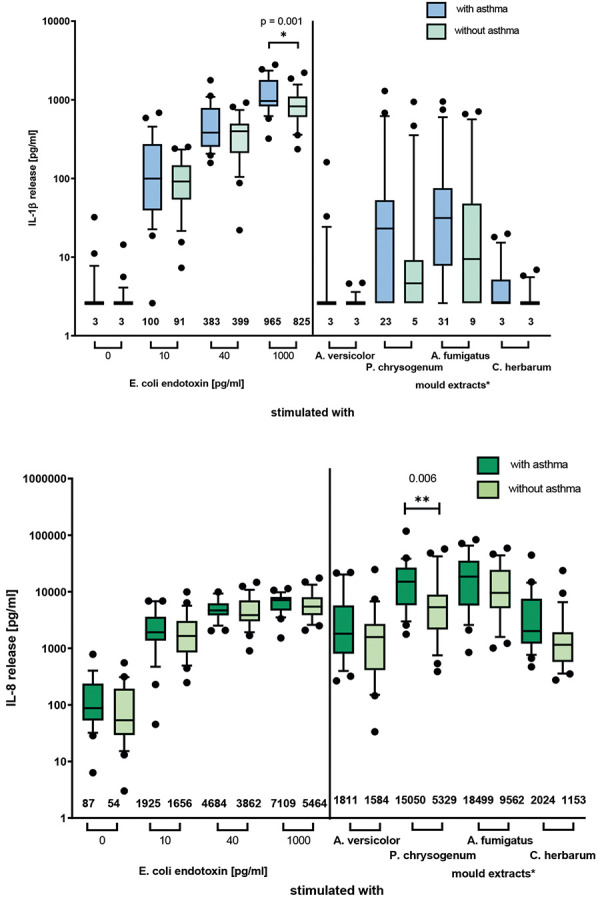
Comparison of cytokine release in whole blood assay of asthmatics (n = 24) and non-asthmatics (n = 24) with respect to IL-1β and IL-8 release after stimulation with endotoxin or mold extracts. Median and 90% percentile are shown. *Molds were used in an estimated final concentration of 1 mg/mL based on calculation of the total extracted weight of mold (30 mg/mL).

The asthmatic group included a higher portion of males (62 vs. 42%) and subjects above 50 years of age (54 vs. 33%), while there were no differences in smoking habit. The proportion of mx1-positive subjects was significantly higher in those with asthma compared to the non-asthmatics (46% vs. 4%), and the proportion of sx1-positive subjects was also significantly higher among the asthmatics (67 vs. 33%). Mold exposure was documented in 83% of the asthmatics, but only in 37% of non-asthmatics. Regarding WBA (Figure 2), stimulation with 1,000 pg/mL endotoxin resulted in a significantly higher IL-1β release in the asthmatic group. Moreover, IL-8 reactivity induced by *Penicillium chrysogenum* was also significantly higher in the asthmatics. In general, there was a tendency of higher cytokine release in the asthmatic compared to the non-asthmatic group. 

### Characterization of the study group and whole blood assay according to mold-specific IgE 

**Figure 3.**
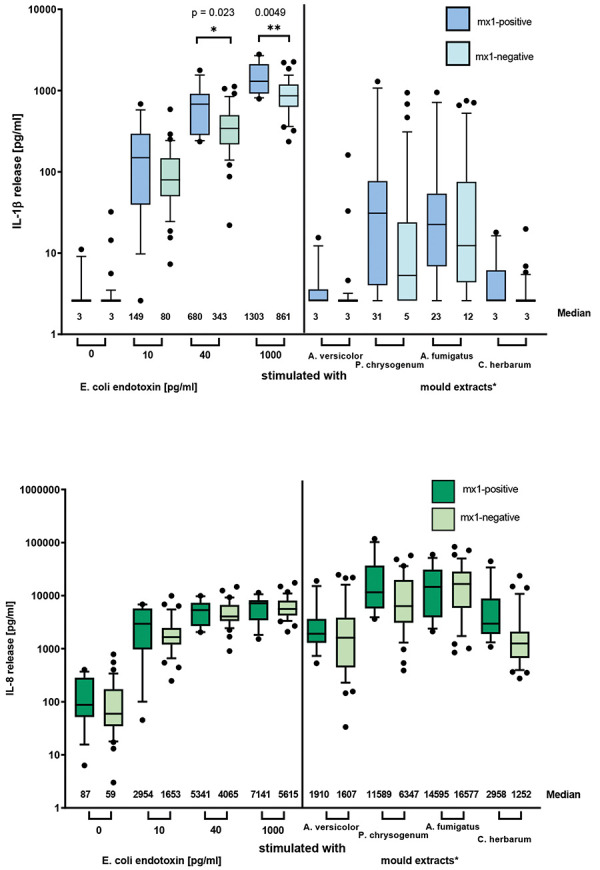
Comparison of cytokine release in whole blood assay of mx1-positive (n = 12) and mx1-negative (n = 36) subjects with respect to IL-1β and IL-8 release after stimulation with endotoxin or mold extracts. Median and 90% percentile are shown. *Molds were used in an estimated final concentration of 1 mg/mL based on calculation of the total extracted weight of mold (30 mg/mL).

Classifying the 48 subjects into either mold-sensitized (sIgE to mx1 ≥ 0.35 kU_A_/L) subjects (n = 12) or non-mold-sensitized (sIgE to mx1 < 0.35 kU_A_/L) subjects (n = 36) showed that respiratory symptoms, including asthma, predominated in the mold-sensitized (mx1-positive) group. Furthermore, 92% of these subjects were sx1-positive compared to 42% in the mx1-negative group (Table 3). With respect to the WBA, a significantly higher IL-1β- release was documented in the mold-sensitized group after stimulation with 40 and 1,000 pg/mL endotoxin. Although no significant difference was observed between the two groups when stimulated with the mold extracts, we observed a tendency towards higher IL-1β release in the mold-sensitized compared to the non-mold-sensitized subjects when stimulated with *Penicillium chrysogenum* and *A. fumigatus* (Figure 3). No significant difference in IL-8 was detectable between both groups after stimulation with either endotoxin or the different mold extracts. 

## Discussion 

Based on our previous research with the WBA [8, 9, 13, 15], we designed the present pilot study to investigate whether using the WBA with fresh blood samples can be useful as a complementary diagnostic tool to better detect a mold-exposed individual. The pyrogenic and pro-inflammatory in vitro response to *Escherichia coli* endotoxin and to four mold extracts was analyzed and evaluated using both questionnaire and serological data for individual strain measurement. 

Although the level of in vitro cytokine response was not significantly correlated with the intensity of mold exposure in this study group, associations between in vitro cytokine response and asthmatic symptoms, as well as mold sensitization (mx1 sIgE ≥ 0.35 kU_A_/L) were observed. Extracts from the mold species *Aspergilus fumigatus* and *Penicillium chrysogenum* were most effective in inducing cytokine release after in vitro stimulation, regardless of the documented exposure. 

IL-8 release was triggered in the entire study group with all four mold extracts. Furthermore, reactivity to the extracts from *Aspergillus fumigatus* and *Penicillium chrysogenum* were highest with respect to IL-8 as well as IL-1β release. The majority of subjects (71%, 34 out of 48) produced a higher IL-8 release in the WBA by stimulation with the *Aspergillus fumigatus* extract compared to endotoxin stimulation. This is in accordance with data from Oya et al. [16] who showed *Aspergillus fumigatus* to be the most potent species regarding inflammatory potential in their in vitro experiments. Furthermore, Oya et al. [16] confirmed that spores are in general less proinflammatory than hyphae. Since we did not examine the percentage of hyphae and spores in the mold extracts used in our study, we cannot exclude the possibility that different compositions may influence the results. Furthermore, Baxi et al. [3] reported that *Aspergillus* and *Penicillium* are the most common indoor fungal genera, and consequently, could be the most important stimulators of the immune system. 

As outlined by Kespohl et al. [12], the anamnestic estimation of mold exposure is difficult because duration, intensity, and actual mold trigger can vary according to the individual. In addition, the health status also varies among subjects. The synergistic effects of molds with other microorganisms should also be considered, especially since the health effects triggered by microbes may vary at species level, contributing to the complex situation of fungal and bacterial exposure [17]. Punsmann et al. [8] described that the in vitro response to *Aspergillus fumigatus* could be synergistically increased by adding small amounts of endotoxin. Thus, immune responses can be influenced by numerous factors, both humoral and cellular, and further research is needed to better understand the contribution of the various factors. 

Punsmann et al. [8] described an increased IL-1β release in WBA with fresh blood from mold/dampness-exposed subjects after stimulation with *Escherichia coli* endotoxin, whereas the IL-8 and TNF-α release were not affected. Furthermore, it was described that stimulation with *Aspergillus versicolor* extract induced a significant increase in IL-1β and IL-8 release in the WBA from exposed subjects. These results could not be confirmed in the present study. In contrast, the experiments presented here suggest that the *Aspergillus versicolor* extract was the weakest stimulus for cytokine release in all subjects tested, although the same extraction protocol was used as described by Punsmann et al. [8]. Considering that the precise composition of these mold extracts is unknown, natural variation of this biological material may play a pivotal role in the reactivity of the molds in WBA. Furthermore, individual diversity of cytokine responsiveness in the WBA has already been demonstrated in several studies [13, 15, 18]. In addition, other molds may be of importance in occupationally exposed subjects than in the context of private households, as described by Kespohl et al. [12] 

Nevertheless, Spierenburg et al. [19] suggested that the WBA should be considered as a viable tool to investigate associations with current health status as well as exposure, especially with IL-1β as a stable marker. Indeed, the current study confirmed higher IL-1β release for the mold-sensitized (mx1-positive) and the asthmatic sub-group, but not in association with mold exposure. These results show that it is more likely that disease-related changes in the innate immune response can be described using the WBA, while it is more difficult to represent only the effects of exposure before health effects occur. 

Although we were able to show in general that molds are suitable stimuli for innate immune responses and that the *Aspergillus fumigatus* extract was the most effective elicitor of a cytokine response in the WBA of the four extracts investigated, in this pilot study, we were unable to identify any reasons for the different reactivity of the mold extracts in the WBA. Warris et al. [20] showed that conidia and hyphal fragments of *A. fumigatus* stimulated cytokine release in whole blood from healthy donors. *Aspergillus fumigatus* hyphae, as the invasive form, appeared to stimulate the release of the proinflammatory cytokines, TNF-α and IL-6 more strongly than the non-viable conidia. Warris et al. [20] discussed a dysregulation between pro- and anti-inflammatory cytokines as a pathogenic alteration of the immune system due to fungal stimulation. Thus, measurement of further cytokines, additional biomarkers and defense mechanisms of other cells like e.g. reactive oxygen intermediates from polymorphonuclear cells may be needed in order to fully describe the effects of mold exposure. In addition, experiments testing the outcome of simultaneous stimulation with mold and endotoxin may also be helpful in better understanding the possible synergistic effects of mold with further microbial exposure on the innate immune system [8]. 

Smit et al. [21] stated that ex vivo cytokine release may reflect sensitivity to occupational endotoxin exposure. The authors found significantly positive dose-response relationships between endotoxin exposure and asthma symptoms in subjects with higher cytokine release, as well as significant associations between endotoxin exposure and a lower forced expiratory volume (FEV_1_). In the present study, we also investigated the associations between mold exposure in vivo and the ex vivo/ in vitro cytokine response. However, a major difference to our study is that no mold measurements were carried out for exposure assessment in our study, as the exposure data are based on the information provided in the questionnaire. Furthermore, the group was too small to adjust for dampness or extensively exposed subjects. It is possible that studies with a larger number of blood samples that allow a more detailed and evidence-based classification (e.g., based on exposure assessment) could provide more detailed information. 

It is known that age and gender may influence cytokine release. Von Aulock et al. [22] showed that blood from male subjects elicited a stronger response in the WBA induced by lipopolysaccharide or lipoteichoic acid and released more TNF-α, IL-1β, IL-6, and IL-8 than blood from female subjects. In our study group, these factors could be excluded as main drivers, as age and sex did not differ significantly between the exposed and non-exposed groups. Furthermore, the influence of smoking could not be assessed, as only 8 of the 48 study participants were smokers. 

Microbial exposure and the interaction with the immune system plays a central role in human health [23]. Aerosolized particles may range from cotton dust to fungal hyphae or bacterial cells, as well as their fragments or aggregates [24]. In comparison to other environmental allergens, the sensitizing potential of molds is estimated to be low, with a prevalence of sensitization of 3 – 10% reported among the total European population [1]. In addition to the IgE-mediated mechanism of sensitization, other reaction pathways in the immune system are possible that are triggered by molds. 

An explanation for the low sensitization rate by molds may be hydrophobin, a surface layer on the dormant conidia that masks their recognition by the immune system [25]. Thus, recognition of molds follows other rules than e.g. that of pollen. 

Whether an induced immune reaction, which primarily serves to protect against a foreign substance/pathogen, turns into a pathological condition certainly depends on many factors that can vary greatly from individual to individual but the level and duration of mold exposure being the starting point. Therefore, from a preventive medical point of view, any mold damage that has occurred should be eliminated as soon as possible [1]. 

In summary, in vitro stimulation of fresh blood from individual subjects with mold extracts or endotoxin and the subsequent cytokine release cannot be recommended for validation of mold exposure in routine diagnostics. However, in combination with other methods, WBA helps to accurately describe the complex and individual immune response to molds. 

## Acknowledgment 

We would like to thank the medical-technical colleagues from the Clinical Center and Department of Allergology/Immunology for their valuable work and the scientific colleagues for their constructive comments as well as Dr. Rosemarie Marchan for improving the language. 

## Funding 

The study was supported by the DGUV (German Social Accident Insurance, Sankt Augustin, Germany, IPA-project 145 Bioaerosole). 

## Conflict of interest 

The authors declare no conflict of interest. 

**Table 1.** Classification of all subjects (n = 48) based on their mold exposure.

Whole blood assay study group (n = 48)		
	Mold-exposed (n = 29)	Non-mold exposed (n = 19)
Male: n (%)	16 (55)	9 (47)
Age (years): median (range)	48 (23 – 75)	45 (22 – 60)
≥ 50 years: n (%)	13 (45)	8 (42)
Smoker: n (%)	5 (17)	3 (16)
Mold-sensitized (mx1): n (%)	10 (34)	2 (11)
sx1-positive: n (%)	18 (62)	8 (42)
Respiratory symptoms: n (%)	26 (90)	9 (47)
Asthma: n (%)	20 (69)	4 (21)

**Table 2.** Classification of all subjects (n = 48) based on questionnaire documented asthma diagnosis.

Whole blood assay study group (n = 48)		
	With asthma (n = 24)	Without asthma (n = 24)
Male: n (%)	15 (62)	10 (42)
Age (years): median (range)	50 (23 – 75)	44 (22 – 60)
≥ 50 years: n (%)	13 (54)	8 (33)
Smoker: n (%)	4 (17)	4 (17)
Mold-sensitized (mx1): n (%)	11 (46)	1 (4)
sx1-positive: n (%)	16 (67)	8 (33)
Respiratory symptoms: n (%)	24 (100%)	11 (46)
Mold exposed: n (%)	20 (83)	9 (37)

**Table 3.** Classification of all subjects (n = 48) based on mold sensitization (mx1-sIgE ≥ 0.35 kUA/L).

Whole blood assay study group (n = 48)		
	Mold sensitized (mx1) (n = 12)	Non-mold sensitized (n = 36)
Male: n (%)	10 (83)	14 (39)
Age (years): median (range)	52 (23 – 65)	46 (22 – 75)
≥ 50 years: n (%)	7 (58)	14 (39)
Smoker: n (%)	2 (17)	6 (50)
Respiratory symptoms: n (%)	12 (100)	23 (64)
Asthma: n (%)	12 (100)	13 (36)
Mold-exposed: n (%)	10 (83)	19 (53)
sx1-positive: n (%)	11 (92)	15 (42)
